# [Corrigendum] Expression of family with sequence similarity 172 member A and nucleotide‑binding protein 1 is associated with the poor prognosis of colorectal carcinoma

**DOI:** 10.3892/ol.2023.13821

**Published:** 2023-04-18

**Authors:** Wenjun Liu, Shuang Wang, Kai Qian, Jinqian Zhang, Zhi Zhang, Hao Liu

Oncol Lett 14: 3587–3593, 2017; DOI: 10.3892/ol.2017.6585

Following the publication of this article, an interested reader drew to the authors’ attention that the immunohistochemical images shown in Fig. 1C and F on p. 3590, showing the results of high FAM172A and high NUBP1 expression levels in CRC tissue samples respectively, appeared to be overlapping, such that the data were likely to have been derived from the same original source. The authors were able to examine their original data, and realized that an incorrect data panel was selected for Fig. 1F.

The revised version of Fig. 1, now showing the correct data for Fig. 1F, is shown on the next page. Note that this error did not greatly affect either the results or the conclusions reported in this paper, and all the authors agree to the publication of this Corrigendum. The authors regret that this error went unnoticed in the published version of their article, and apologize to the readership for any inconvenience caused.

## Figures and Tables

**Figure 1.**
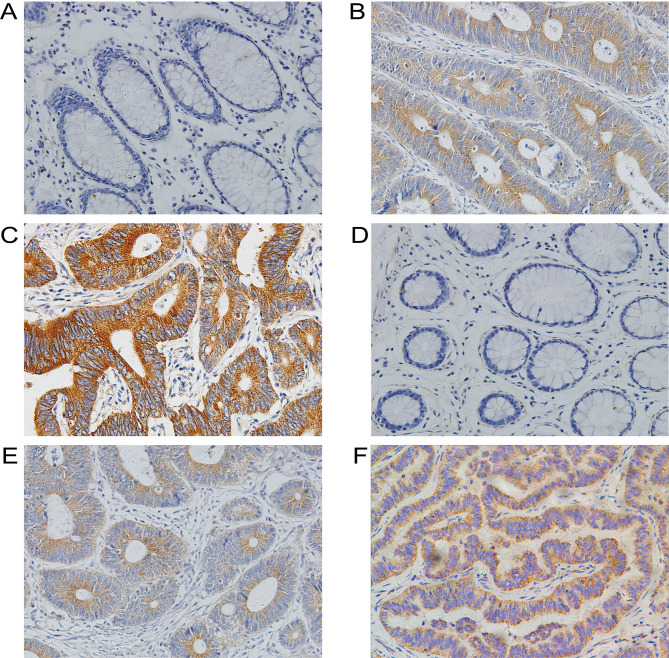
Immunohistochemical staining in CRC. (A) Normal colorectal mucosa was negative for FAM172A expression. (B) Low FAM172A expression level in CRC tissue samples. (C) High FAM172A expression level in CRC tissue samples. (D) Normal colorectal mucosa was negative for NUBP1 expression. (E) Low NUBP1 expression level in CRC tissue samples. (F) High NUBP1 expression level in CRC tissue samples (magnifications, ×200). NUBP1, nucleotide-binding protein 1; FAM172A, family with sequence similarity 172 member A; CRC, colorectal cancer.

